# Perforator propeller flaps for sacral and ischial soft tissue reconstruction

**DOI:** 10.4103/0970-0358.73427

**Published:** 2010

**Authors:** Pradeoth M. Korambayil, KV Allalasundaram, TM Balakrishnan

**Affiliations:** Department of Plastic Reconstructive and Aesthetic Surgery, Madras Medical College, Chennai; 1 Department Plastic and Reconstructive Surgery, Madras Medical College, Chennai

**Keywords:** Parasacral perforator-based flap, pilonidal sinus, propeller flaps, sacral and ischial pressure sore, superior and inferior gluteal artery perforator-based flaps

## Abstract

The perforator-based flaps in the sacral and ischial region is designed according to the localization of perforators that penetrate the gluteus maximus muscle, reach the intra-fascial and supra-fascial planes with the overlying skin forming a rich vascular plexus. The perforator-based flaps described in this article are highly vascularized, have minimal donor site morbidity, and do not require the sacrifice of the gluteus maximus muscle. In a period between April 2008 and March 2009, six patients with sacral pressure sore were reconstructed with propeller flap method based on superior gluteal and parasacral artery perforators. One flap loss was noted. Three cases of ischial pressure sore were reconstructed with longitudinal propeller flap cover, based on inferior gluteal artery perforator. One flap suffered wound infection and dehiscence. Two cases of pilonidal sinus were reconstructed with propeller flap based on parasacral perforators. Both the flaps survived without any complications. Donor sites were closed primarily. In the light of this, they can be considered among the first surgical choices to re-surface soft tissue defects of the sacral and ischial regions. In the series of 11 patients, two patients (18%) suffered complications.

## INTRODUCTION

Sacral and ischial soft tissue defects have always been a challenge to the plastic surgeon. Numerous surgical techniques are employed in their repair and each has its own advantages and disadvantages. Pressure sore and pilonidal sinus are common conditions resulting in sacral and ischial soft tissue defects. We present 11 patients operated on for sacral, ischial pressure sores and pilonidal sinus using perforator-based flaps.

## PATIENTS AND METHODS

The study was conducted in the Department of Plastic Surgery, Government General Hospital, and Madras Medical College. Eleven patients, eight males and three females, were operated upon over a period of 12 months (April 2008 to March 2009). The mean age of the group was 37 (age range: 22–50 years). Dimensions of the skin defects and operative details for all patients are shown in Table 1.

**Table 1:** Dimensions of the skin defects and operative details for all patients

*No:*	*Age/Sex*	*Diagnosis*	*Localization*	*Defect Size (cm)*	*Flap Size (cm)*	*Details*	*Origin of perforators (No. of Perforators)*	*Rotation degree*	*Complication*
1	26/M	Ischial pressure sore(r)	Ischial	5×5	6×7	Lumbar meningomyelocele operated at 40 days of age	Inferior gluteal A r Perforator 1 AV	40	nil
2	45/M	Grade IV sacral pressure sore	Sacral	11×8	12×9	Seizure disorder anterior decompression cervical spine	Parasacral A r Perforator 2 AV	90	nil
3	50/M	Grade IV sacral sore	Sacral	6×5	7×6	#D12 with paraplegia anterior decompression done	Parasacral A r Perforator 1 AV	90	nil
4	50/F	Intergluteal pilonidal sinus	Sacral	7×6	8×7	Discharging sinus intergluteal cleft	Parasacral A r Perforator 1 AV	90	nil
5	45/M	Sacral sore (left side)	Sacral	5×6	6×7	C4C5 subluxation with Quadriplegia	Superior gluteal A r Perforator 1 AV	90	nil
6	37/M	Grade IV lschial pressure sore	Ischial	7×6	8×7	Healed sacral sore, rotation flap for ischial sore with remnant sore	Inferior gluteal A r Perforator 1 AV	90	Wound dehiscence
7	30/F	Intergluteal pilonidal sinus	Sacral	9×4	10×5	Discharging sinus intergluteal cleft	Parasacral A r Perforator 1 AV	90	nil
8	45/M	GradeIV sacral sore	Sacral	13×10	14×11	# D12 with paraplegia - anterior decompression done	Superior gluteal A r Perforator 1 AV	180	nil
9	28/F	Grade IV lschial pressure sore	Ischial	7×4	8×5	# D12 anterior decompression, posterior thigh flap done for contralateral ischial sore	Inferior gluteal A r Perforator 1 AV	90	nil
10	22/M	Grade IV sacral sore	Sacral	9×7	10×8	Multiple punctate contusion left parietal brain	Superior gluteal A r Perforator 1 AV	110	Total flap loss
11	25/M	Grade IV sacral sore	Sacral	9×7	10×8	#D1 D8 paraplegia	Superior gluteal A r Perforator1 AV	110	nil

**Figure 1:**
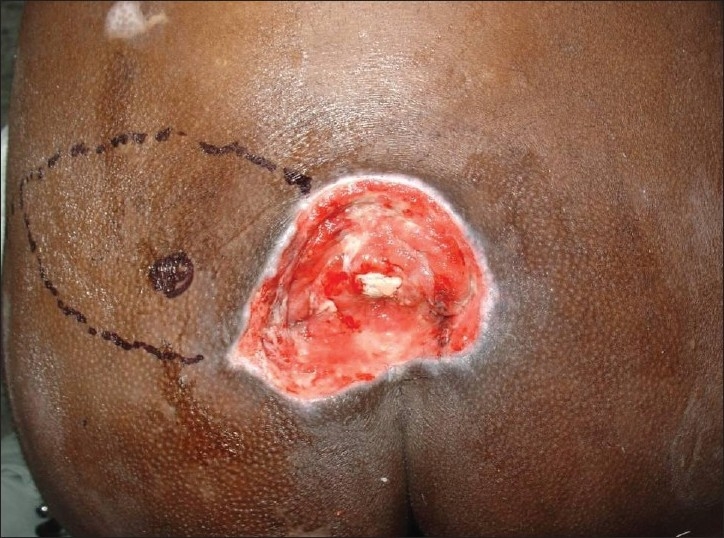
Grade-IV sacral pressure sore

**Figure 2:**
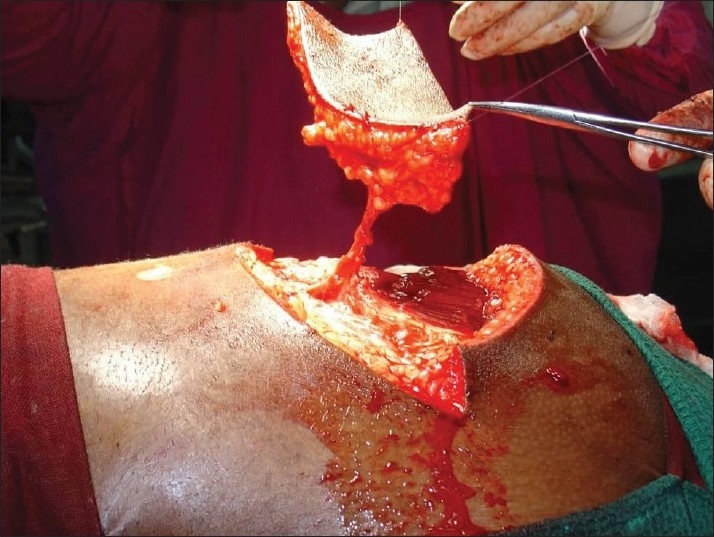
Pressure sore reconstructed with superior gluteal artery perforator

Five (45.4%) patients were paraplegic, four (36.36%) were ambulatory and one (9%) patient was quadriplegic. The ambulatory patients presented with pressure sores resulting from prolonged immobilization after surgery. Of those sores, six (54.5%) were sacral, three (27.2%) were ischial and two (18.1%) were pilonidal sinus. Flaps with dimensions of 6 × 7 cm^2^–14 × 11 cm^2^ were designed. Flaps were mostly raised based on one or two perforators at least [Figure 1]. Only one flap was transposed based on two perforators [Figure 2]. Remaining defects were repaired using perforator-based flaps with one perforator.

## SURGICAL TECHNIQUE

Dimensions of the skin defect were recorded. The localization of perforators around the sacrum was pre-operatively determined by a handheld Doppler ultrasound scanner. A provisional flap design would be then drawn. Acentric axis type, where the pedicle was located on an acentric portion of the flap that can rotate 180 degrees and cover skin defects at some distance, was planned. Distance between the perforator and the distal edge of the defect was measured. This value was then transposed proximally, again measured from the perforator, and one centimetre would be added to it to form the proximal limit of the flap. The width of the defect was measured and half a centimetre was added to it. All cases were operated under loupe magnification (4.5×). The perforator vessels were located through an exploratory initial incision. The approach to the pedicle would be sub-fascial. With this initial incision, a number of potentially useful perforators, based on its position, size and presence of concealed injury to the pedicle, were exposed. A visual assessment of the perforators was then made to choose the best pedicle for the flap. When the perforator was finally chosen, re-designing and adjustment of flap dimension was carried out, if necessary. Careful dissection around the pedicle was done to clear off all muscular side branches from its vessel of origin to the point where the pedicle penetrated the deep fascia of the flap or for at least 2 cm. Meticulous division of all the fascial strands using magnification that would potentially cause vascular embarrassment through kinking of the vessels was performed. Once the pedicle was secured, rest of the flap would be raised. The flap was completely islanded. It was left in its original position for 10–15 minutes to allow it to perfuse and to allow the spasm of the vessels to relax. Topical vasodilators were instilled around the pedicle at this point. Once the flap perfusion was satisfactory, the flap was carefully lifted from the donor bed and rotated around this pedicle into the recipient defect. Rotation can vary, from say 90° to a maximum of 180°, looking in particular for any sign of kinking by any residual fascial strands that may need further division. The flap was secured in the desired position with the first two skin sutures placed on either sides of the axis of the pedicle; ensuring that the pedicle was not put under any traction tension either in a proximal or distal direction. A suction drain was placed carefully under the flap and secured well away from pedicle. The rest of the flap inset and wound closure was then completed. The donor defect was closed primarily or by local flaps. Patients were nursed in prone or in lateral position to offload the reconstructed area.

## RESULTS

Six patients with sacral pressure sore were reconstructed with perforator-based propeller flap method based on superior gluteal artery perforator. Five among these six flaps survived completely. Donor area was closed primarily in five cases. In one case with flap dimension of 14 × 11 cm, donor area could not be closed, primarily necessitating a local rotation flap for closure of the donor defect. Three cases of ischial pressure sore were reconstructed with perforator-based propeller flap cover based on inferior gluteal artery perforator. Two among these three flaps survived completely. Donor area was closed primarily. Two cases of pilonidal sinus after thorough debridement were reconstructed with longitudinal propeller flap based on superior gluteal artery perforators. Both the flaps survived without any complications. Donor sites were closed primarily.

One case of sacral pressure sore, reconstructed by superior gluteal artery perforator-based flap developed respiratory distress in the immediate post-operative period and was managed on mechanical ventilator. Patient developed alterations in metabolic parameters and difficulty in maintaining the position post-operatively. The flap showed persistent congestion and later, we had a total flap loss. A case of ischial pressure sore reconstruction with inferior gluteal artery perforator-based flap got infected and there was wound dehiscence, which was later managed by local rotation flap.

## CASE REPORTS

### Case 1

**Figure 3:**
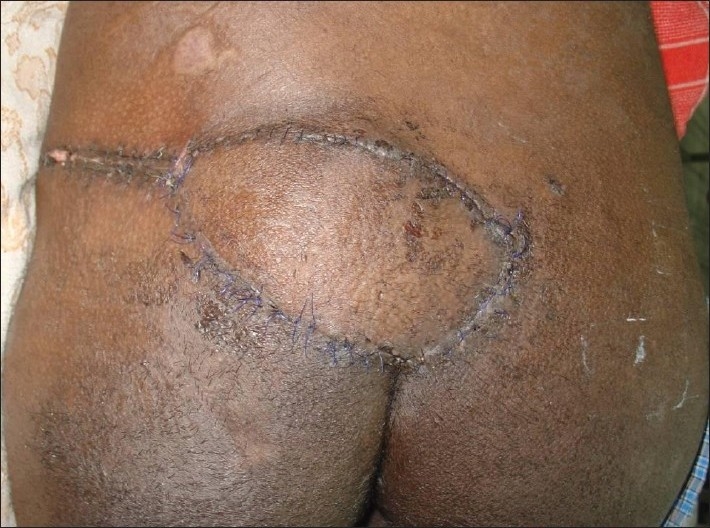
Late post-operative picture

A 25-year-old paraplegic male patient was referred with grade IV sacral pressure sore [Figure 1]. The sacral defect was reconstructed using a superior gluteal artery perforator-based propeller flap [Figure 2]. There were no post-operative complications [Figure 3].

### Case 2

**Figure 4:**
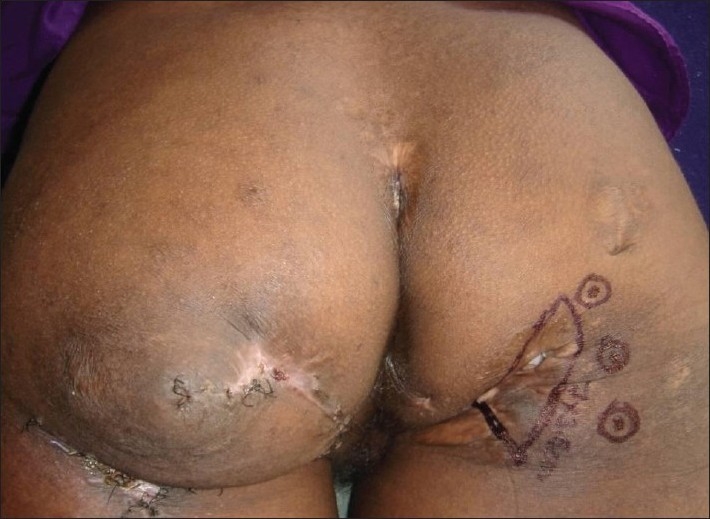
Grade-IV ischial pressure sore, contralateral ischial pressure sore reconstructed with posterior thigh flap previously

**Figure 5:**
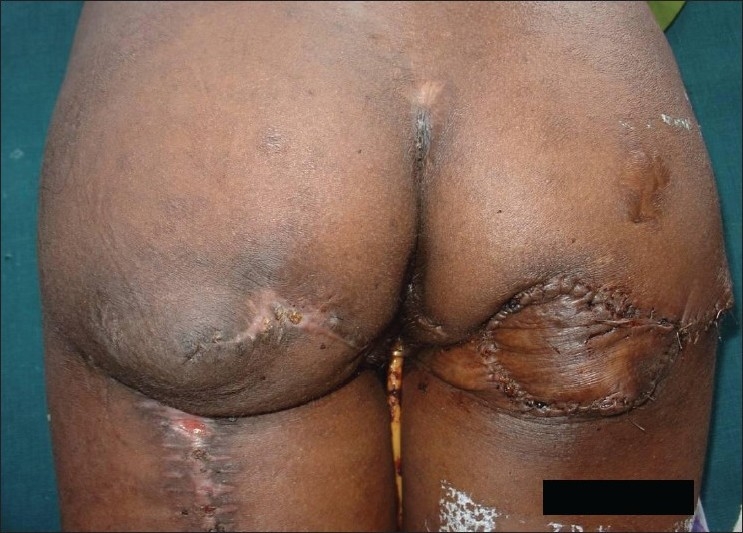
Defect reconstructed with inferior gluteal artery perforator-based propeller flap

A 28-year-old paraplegic female patient was referred with grade IV ischial presssure sore on the right side [Figure 4]. The patient was previously operated for contralateral ischial pressure sore with posterior thigh flap. Inferior gluteal artery perforator-based propeller flap was used for the reconstruction. Flap healed without any complication [Figure 5].

### Case 3

**Figure 6:**
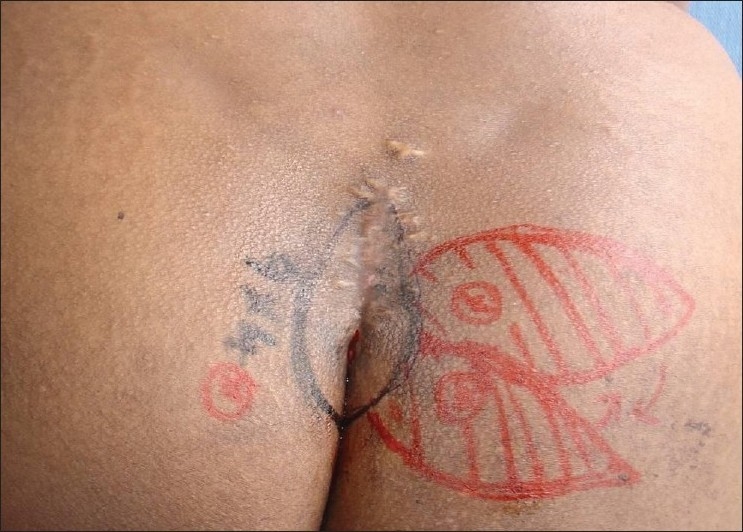
Intergluteal pilonidal sinus reconstructed with parasacral perforator

**Figure 7:**
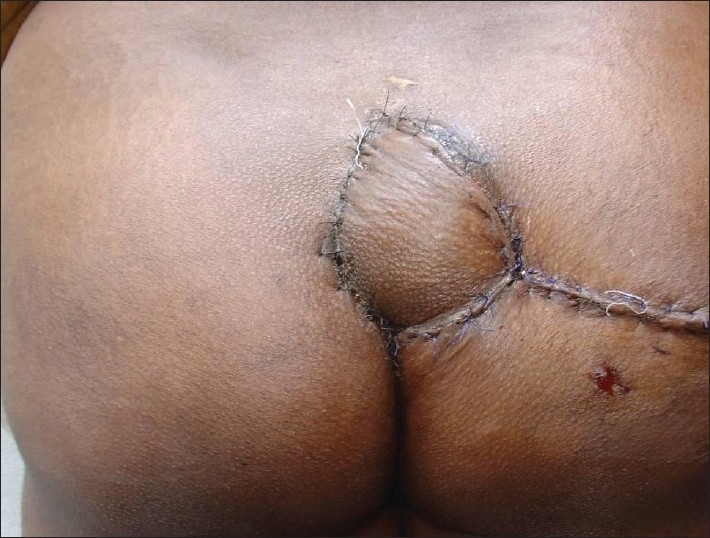
Post-operative picture

A 30-year-old female patient with intergluteal pilonidal sinus [Figure 6] was managed by wound debridement and the defect was reconstructed with superior gluteal artery perforator-based propeller flap. Flap recovery was uneventful [Figure 7].

## DISCUSSION

Pressure-sore defects present a difficult challenge because of the high rates of wound complications and recurrences. Myocutaneous flaps have been considered as the standard first-line treatment for pressure sores that fail conservative therapy. These flaps were transposed as a rotational flap,[1] as an island flap[2] or as a V-Y advancement flap.[3–5] These flaps are reliable because of an abundant blood supply. However, all these flaps were elevated away from the underlying gluteus medius muscle. In the gluteus maximus musculocutaneous V-Y advancement flap, the origin was detached. When necessary, the insertion of the gluteus maximus muscle was divided.[6] The motor innervation of the gluteus maximus muscle is by means of the inferior gluteal nerve and parallels the course of the inferior gluteal artery. The gluteus maximus muscle extends and laterally rotates the hip joints, and the lower fibres also assist in adduction of the hip joints. The upper fibres assist in abduction. By its insertion into the iliotibial tract, it helps to stabilize the knee in extension. A bilateral marked weakness of the gluteus maximus makes walking extremely difficult in an ambulatory patient and necessitates the aid of crutches.[7] The bilateral superior halves of the gluteus maximus muscle, with an overlying skin island, are released from its origin and insertion. Thus the inferior half of each gluteus maximus is preserved, avoiding hip instability.[8] Muscle sparing should be considered in paraplegic patients as well. Limitation of sliding gluteus maximus muscle cover as described by Ramirez *et al*.[5] in 1984 includes: increased blood loss, the increased operating time and the tension on the edges of the flaps, sacrifice of gluteus maximus muscles, which results in loss of the future reconstructive possibilities. The preservation of muscle integrity and muscle function is one of the greatest assets of the perforator flap principle. Especially in non-paralysed patients who will need full function of the gluteal muscles for recovery of ambulation, the knowledge that function is kept intact may significantly lower the threshold towards decubitus reconstruction with good-quality tissue. Sacrifice of underlying muscle is required in the inferior gluteal myocutaneous rotation flap, a commonly used means of ischial reconstruction in these patients. The donor-site dissection requires closure over the dead space created by the disinserted muscle. We have observed that this is a common site of post-operative wound breakdown after this reconstruction. The perforator counterpart permits tension-free donor-site closure over an intact muscle bed. Myocutaneous flaps for ischial reconstruction often leave re-advancement of the failed flap as the only means of addressing recurrence. The inferior gluteal artery perforator flap spares all muscle and myocutaneous flaps for future use, if required. A myocutaneous flap has been used routinely for reconstructing pressure sores in the pelvic regions on account of its good vascularity. Yamamoto[9] found that fasciocutaneous flaps were expected to provide better long-term results in surgical reconstruction of pressure sores than the myocutaneous or muscle flap.

The advantages of the perforator flap over the traditional flap include reduced bleeding, preservation of the muscle and its function, versatility of the flap design to yield a better match to the defect and increased arc of rotation of the flap.

**Figure 8:**
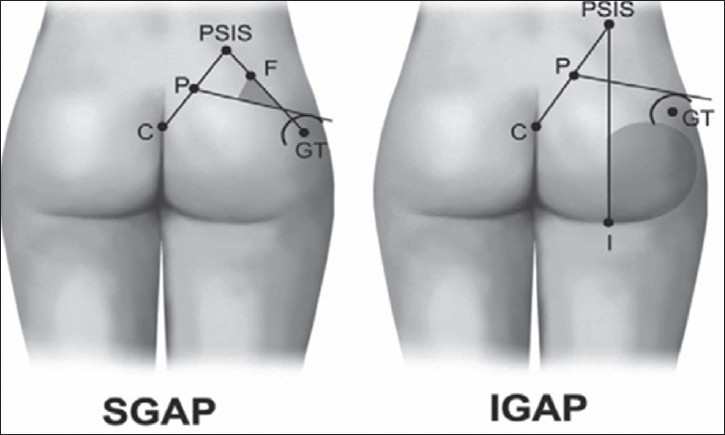
Schematics for facilitating the design of the superior gluteal artery perforator (SGAP) and inferior gluteal artery perforator (IGAP) flaps in gluteal region.

Disadvantage of perforator propeller flap is that the flaps are mostly insensate. Most non-innervated local flaps as described by Teo (personal communication), eventually develop a slight sensory recovery due to peripheral innervations. However, a myocutaneous flap is still a better choice when filling an extensive cavity with adequate bulk is indicated. A cadaveric study disclosed the existence of several perforators all around the gluteal region.[10] Several main perforators of large calibre were found in the parasacral and central portions of the gluteal muscle. These significant perforators pass through the muscle itself and the fascial portion of the muscle to the overlying skin on the gluteal region. In 1993, Koshima *et al*.[10] published their early results with gluteal perforator-based flaps for repair of sacral pressure sores. Majority of Koshima’s patients received a flap based on several perforators, which needed to be rotated over 60–180° to cover the defect. Kroll and Rosenfield[11] reported a flap based on unnamed perforators located near the midline of the lower-back region to repair lower-posterior midline-defects. In this method, muscle function is preserved, but complete skeletonization of the perforators is needed to allow mobilization of the flap. Four of our initial flaps were based on gluteal perforators in the parasacral region, four flaps were based on superior gluteal artery perforator and three flaps were based on inferior gluteal artery perforator. Pre-operative Doppler flowmetry is often used to rapidly identify the perforating vessels in the anatomical area of interest. However, the procedure is operator dependent, time-consuming, and not always accurate in localizing the perforating vessels.[12] Therefore, Doppler evaluation could be limited to confirm intra-operatively the choice of the perforator vessel performed under direct visualization during the dissection. Other imaging methods (Doppler ultrasound[12] and multislice CT angiography[13]) are used worldwide to pre-operatively localize the vessels in perforator flap surgery. In one patient, two arteriovenous perforators could be included in the flap as there was lesser degree of rotation needed. The flaps taken from the parasacral area have a risk of perforators nearer to the injured zone. All perforators were dissected through the muscle to obtain adequate length but there was no need to dissect unto the source vessel. Average calibre of parasacral perforators were 1–2 mm and those of superior and inferior gluteal perforators were 3–4 mm. Blondeel PN and Hallock GG[14] recently described a primer of schematics [Figure 8] for facilitating the design of the superior gluteal artery perforator (SGAP) and inferior gluteal artery perforator (IGAP) flaps in gluteal region. In gluteal region, SGAP lines are drawn from the posterior superior iliac spine (PSIS) to the coccyx (C), and to the apex of the greater trochanter (GT). A line from the midpoint (P) of PSIS-C to the superior edge of the greater trochanter corresponds to the course of the piriformis muscle. Point F at the proximal third of the line PSIS-GT corresponds to the exit of the SGA from the pelvis. Perforators will be located in shaded area above the piriformis muscle. For IGAP, lines are drawn from the PSIS to the coccyx (C) and to the ischial tuberosity (I). A line from the midpoint (P) of PSIS-C to the superior edge of the greater trochanter corresponds to the course of the piriformis muscle. Perforators will be located in shaded area below piriformis muscle and above inferior gluteal crease, lateral to the vertical line PSIS-I.

**Figure 9:**
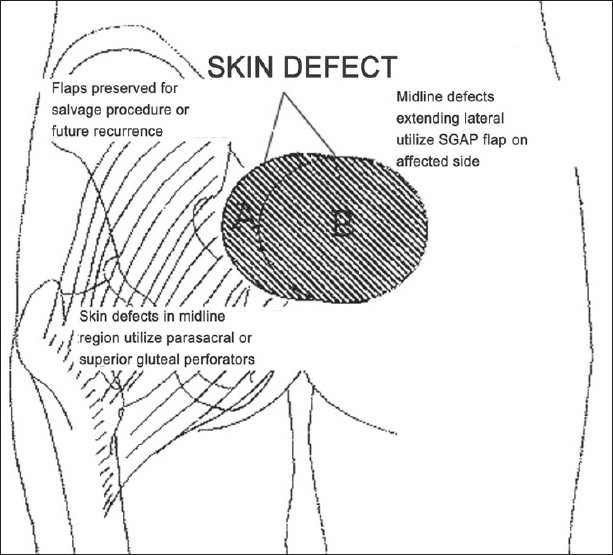
Schematics for choosing the perforator propeller flap considering the possibility of recurrance

**Figure 10:**
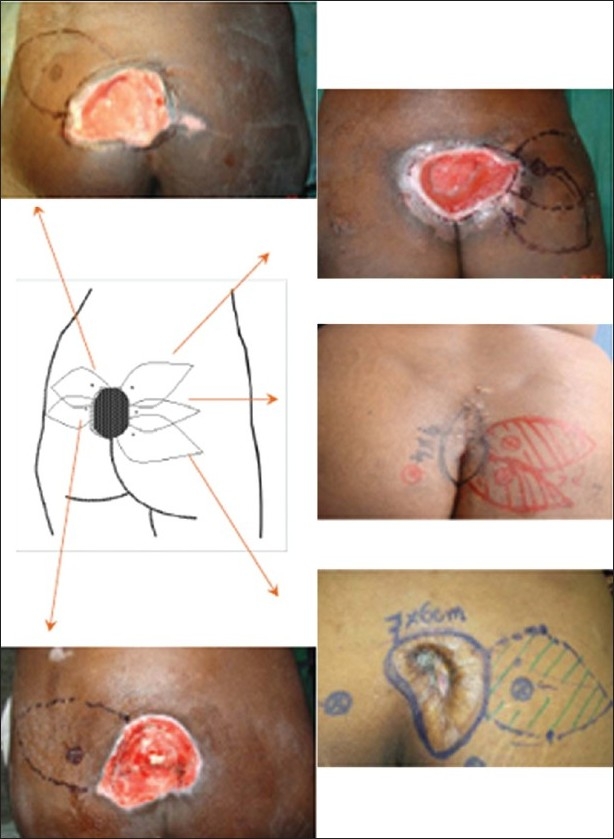
Schematics for choosing the design for the perforator propeller flap to the sacral soft tissue defect

In our present series, the superior and inferior gluteal artery perforators were found in the same site as mentioned above. Design of the propeller flap chosen, considering the possibility of recurrence is represented in the following schematic diagrams [Figures 9 and 10]. Since the perforators travel from medial to lateral direction, with proper skeletonization and mobilization of vessels, the flap could be transferred to the midline defect with much ease compared to perforator propeller flaps in other regions. Verpaele *et al*.[15] described the possibility of raising large skin – subcutaneous flaps – based on one single muscle perforator, at a distance from the injured area. Four of our flaps to cover sacral sores were based on superior gluteal perforator. The dissection of the pedicle takes some time, but is straightforward as it lies in a vascular plane. This gives the additional advantage that the blood loss is kept to a minimum, compared to any gluteus flap of which the dissection of the sacral origin can be quite bloody. Although Meltem *et al*.[16] harvested gluteal perforator flap with maximum dimension of 16 × 20 cm successfully, the largest flap based on gluteal perforators in our study was 14 × 11 cm in dimension. The conservative approach to the flap makes it a safe procedure, even at the beginning of the learning curve. If no perforators had been found at the expected sites, salvage would have been possible with bilateral rotation flaps. The rotation flap suture lines, however, always show some tension and the tip of the flap is less reliable and less bulky than the perforator flap would be. Complete skeletonization of one or two perforators allows wider mobilization of the perforator propeller flap when compared to gluteus V-Y advancement flap as a fasciocutaneous flap with multiple perforators. The superior gluteal artery perforator flap provides us with a large, bulky and safe skin–subcutaneous flap to cover sacral pressure sores. There is no significant donor site morbidity, no bridges are burned and neither muscle nor muscle function is sacrificed. There were no recurrences in the present series during the follow-up period. Coskunfiırat and Özgentas^[17]^ experienced only one recurrence during the 13.6-month follow-up period with 35 gluteal perforator flaps for the coverage of 22 sacral, seven ischial and six trochanteric pressure sores in 32 patients. Soft-tissue integrity depends not only on carefully designed reconstructions but also the patient’s ability to restore blood flow following ischaemia and the avoidance of prolonged pressure.

In conclusion, relatively large defects were reconstructed with single flaps with favourable results. The pedicled perforator propeller flaps of the refined design can be used efficiently in various reconstructions of sacral and ischial defects.

## References

[1] Baek SM, Williams GD, McElhinney AJ, Simon BE (1980). The gluteus maximus myocutaneous flap in the management of pressure sores. Ann Plast Surg.

[2] Maruyama Y, Nakajima H, Wada M, Imai T, Fujino T (1980). A gluteus maximus myocutaneous island flap for the repair of a sacral decubitus ulcer. Br J Plast Surg.

[3] Scheflan M, Nahai F, Bostwick J (1981). Gluteus maximus island musculocutaneous flap for closure of sacral, and ischial ulcers. Plast Reconstr Surg.

[4] Fisher J, Arnold PG, Waldorf J, Woods JE (1983). The gluteus maximus musculocutaneous V-Y advancement flap for large sacral defects. Ann Plast Surg.

[5] Ramirez OM, Orlando JC, Hurwitz DJ (1984). The sliding gluteus maximus myocutaneous flap: Its relevance in ambulatory patients. Plast Reconstr Surg.

[6] Ohjimi H, Ogata K, Setsu Y, Haraga I (1996). Modification of the gluteus maximus V-Y advancement flap for sacral ulcers: The gluteal fasciocutaneous flap method. Plast Reconstr Surg.

[7] Kendall FP, McCreary EK, Provance P (1993). Gluteus Maximus. In Muscle Testing and Function.

[8] Parry SW, Mathes SJ (1982). Bilateral gluteus maximus myocutaneous advancement flaps: Sacral coverage for ambulatory patients. Ann Plast Surg.

[9] Yamamoto Y, Tsutsumida A, Murazumi M, Sugihara T (1997). Long-term outcome of pressure sores treated with flap coverage. Plast Reconstr Surg.

[10] Koshima I, Moriguchi T, Soeda S, Kawata S, Ohta S, Ikeda A (1993). The gluteal perforatorbased flap for repair of sacral pressure sores. Plast Reconstr Surg.

[11] Kroll SS, Rosenfield L (1988). Perforator-based flaps for low posterior midline defects. Plast Reconstr Surg.

[12] Blondeel PN, Beyens G, Verhaeghe R, Van Landuyt K, Tonnard P, Monstrey SJ (1998). Doppler flowmetry in the planning of perforator flaps. Br J Plast Surg.

[13] Alonso-Burgos A, García-Tutor E, Bastarrika G, Cano D, Martínez-Cuesta A, Pina LJ (2006). Preoperative planning of deep inferior epigastric artery perforator flap reconstruction with multislice-CT angiography: Imaging findings and initial experience. J Plast Reconstr Aesthet Surg.

[14] Blondeel PN, Morris SF, Hallock GG, Neligan PC (2005). Perforator flaps: Anatomy, Technique and Clinical Applications.

[15] Verpaele AM, Blondeel PN, Van Landuyt K, Tonnard PL, Decordier B, Monstrey SJ (1999). The superior gluteal artery perforator flap: An additional tool in the treatment of sacral pressure sores. Br J Plast Surg.

[16] Meltem C, Esra C, Hasan, Ali D (2004). The gluteal perforator-based flap in repair of pressure sores. Br J Plastic Surg.

